# Impact of post-vitrification storage duration on clinical outcomes of frozen-thawed cleavage-stage embryos transfer: a propensity score-matched retrospective cohort study

**DOI:** 10.3389/fendo.2026.1765681

**Published:** 2026-04-02

**Authors:** Yunrui Han, Qiyu Yang, Yuxuan Zhao, Yuxin Zhang, Shengyong Ding, Haicui Wu

**Affiliations:** 1 First School of Clinical Medicine, Shandong University of Traditional Chinese Medicine, Jinan, Shandong, China; 2 Department of Pharmacy, Affiliated Hospital of Shandong University of Traditional Chinese Medicine, Jinan, Shandong, China; 3 Department of Reproduction and Genetics, Affiliated Hospital of Shandong University of Traditional Chinese Medicine, Jinan, Shandong, China

**Keywords:** clinical outcomes, embryo vitrification, frozen-thawed embryo transfer, propensity score matching, storage duration

## Abstract

**Purpose:**

To investigate the impact of storage duration after vitrification of cleavage-stage embryos on pregnancy and perinatal outcomes.

**Methods:**

A retrospective study was conducted on 4205 infertile patients underwent frozen-thawed embryo transfer (FET) between January 2020 and January 2025, categorized by post-vitrification storage duration of their embryo: G1 (<3 months, n = 2,208), G2 (3–6 months, n = 1,232), G3 (6–12 months, n = 399), and G4 (>12 months, n = 366). Multivariate logistic and linear regression analyses were used to determine independent effects of storage duration on pregnancy and perinatal outcomes among groups. Propensity score matching was employed to control confounders, including age, infertility duration, type, and etiology.

**Results:**

Regarding pregnancy outcomes, live birth rate significantly decreased with storage duration (using G1 as reference; G2: adjusted odds ratio [aOR] = 0.82, 95% CI [0.707-0.952], similarly hereinafter; G3: 0.743, 0.579-0.954; G4: 0.624, 0.476-0.818). The miscarriage rate significantly increased with increasing storage duration beyond 6 months (G2: 1.268, 0.967-1.663; G3: 1.652, 1.085-2.515; G4: 1.674, 1.06-2.643). Biochemical pregnancy (G2: 0.883, 0.764-1.02; G3: 0.848, 0.669-1.075; G4: 0.705, 0.547-0.91) and clinical pregnancy rates (G2: 0.885, 0.766-1.022; G3: 0.848, 0.669-1.075; G4: 0.715, 0.554-0.922) significantly decreased with increasing storage duration beyond 12 months. Regarding perinatal outcomes, gestational age significantly decreased with increasing storage duration beyond 6 months (G1 as reference; G2: P = 0.128; G3: P = 0.023; G4: P = 0.008).

**Conclusion:**

Overall, prolonged storage duration of vitrified cleavage-stage embryos negatively correlates with pregnancy and perinatal outcomes, particularly after 6 or 12 months.

## 1 Introduction

Embryo cryopreservation is one of the most influential procedures in assisted reproductive technology (ART), and the choice of embryonic stage for cryopreservation significantly affects clinical pregnancy outcomes. Currently, embryonic stage for cryopreservation in clinical is divided into cleavage stage and blastocyst stage. Among these two stages, cleavage-stage embryos exhibit clear developmental potential and is considered preferable for cryopreservation (1). The cleavage stage embryos represent an early phase of development characterized by several mitotic divisions of the fertilized egg, resulting in an increase in cell number while the total volume remains unchanged (2). The spherical and uniformly sized cells with clear cytoplasm and low fragmentation at cleavage stage meets the criteria for a morphologically “high-quality embryo” (3). Based on these criteria, cleavage stage cryopreservation in ART not only allows for accurate morphological scoring but also effective selection of high-quality embryos. Therefore, the cleavage stage has been considered as the optimal window for cryopreservation with extensive clinical application and considerable data for investigating the influence of storage duration on embryonic development and clinical pregnancy outcomes (4).

Embryo cryopreservation technology involves the programmed freezing of embryos to the temperature of liquid nitrogen (−196 °C) to achieve embryo preservation for ART (5). Over four decades, the technology has progressed from slow freezing to widely adopted vitrification (VF) method (6). VF method uses high-concentration cryoprotectants and ultra-rapid cooling, converting both intra- and extracellular fluids directly into a glass-like state without ice crystal formation (7). Compared with slow freezing method, VF markedly improves post-thaw survival and pregnancy outcomes, with procedural simplicity and increased applification (8). Currently, standardized VF protocols and stable techniques have been well established. Despite these advantages, the long-term safety of embryos cryoprotectant concerns persist as a focus of clinical attention and discussion due to the exposure to high cryoprotectant concentrations and liquid nitrogen (9). However, evidence for storage duration influence on maternal or neonatal outcomes remains limited and inconsistent. Some studies report reduced pregnancy rates with longer storage (10), while others find no significant effect (11–14). Potential cytotoxicity and epigenetic effects require further evaluation (15).

Herein, utilizing multi-group propensity score matching (PSM), we analyze the association between storage duration of vitrified cleavage-stage embryos and clinical and perinatal outcomes. We conducted a retrospective study of vitrified-warmed cleavage-stage embryos at our center from January 2020 to January 2025. The purpose is to support embryo bank management and clinical decision-making and to promote standardized ART practice. This study clarifies the impact of VF storage time on pregnancy and perinatal outcomes and provides improved references for clinical practice.

## 2 Materials and methods

### 2.1 Study population

We retrospectively analyzed data from 5,234 frozen-thawed embryo transfer (FET) cycles performed at our center between January 2020 and January 2025. The study included cycles with day-3 cleavage-stage embryo transfer and at least one viable embryo after thawing. Cycles involving blastocyst transfers (n = 760), parental chromosomal abnormalities (n = 29), uterine anomalies (n = 46), endocrine disorders (including diabetes, hypertension, and thyroid disease) (n = 62), and those with incomplete primary outcome data (n = 132) were excluded.

**Figure 1:**
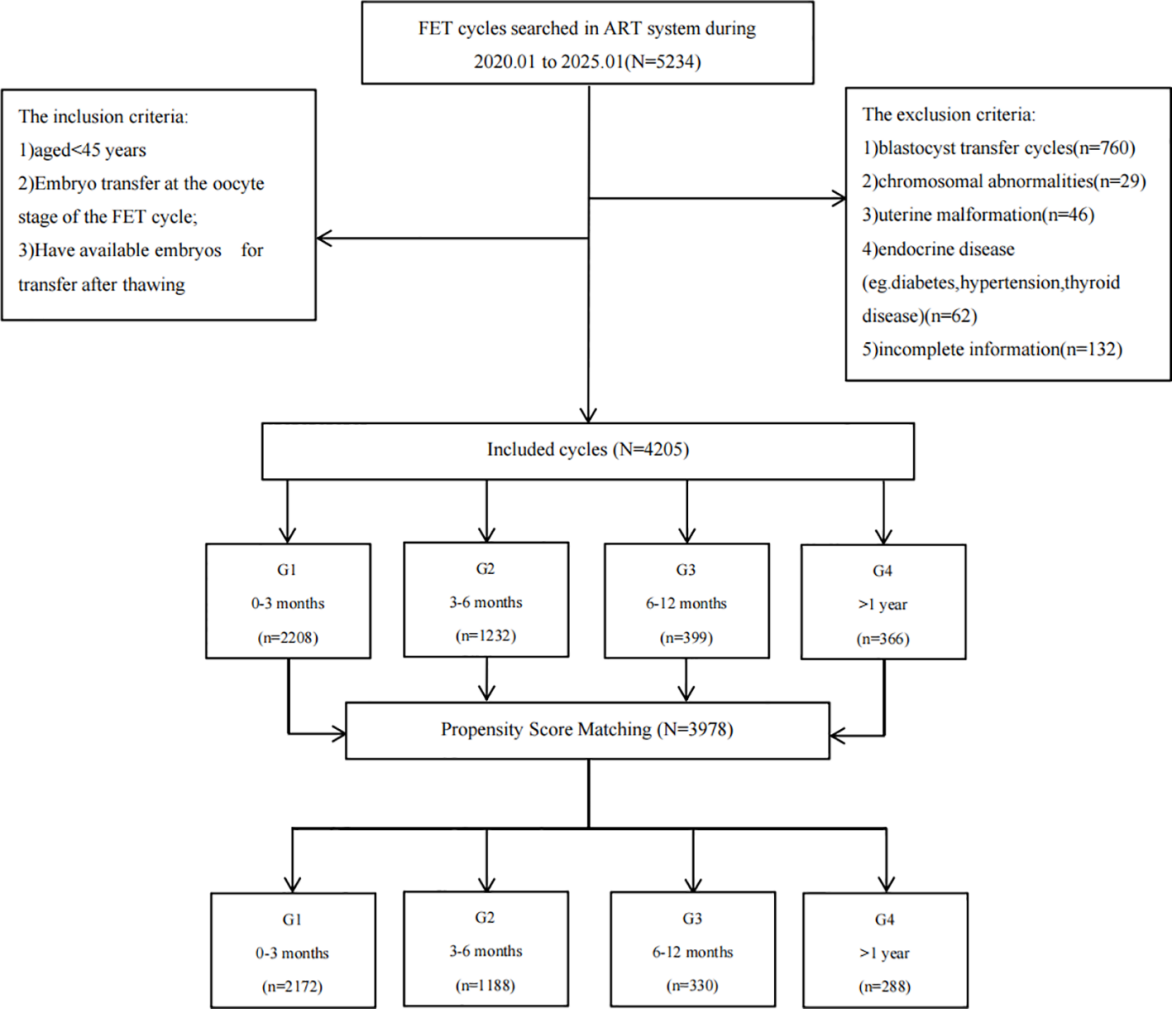
Flowchart illustrating patient selection and PSM.

A total of 4,205 eligible FET cycles were categorized by post-VF storage duration into four groups: Group 1 (<3 months, n = 2,208), Group 2 (3–6 months, n = 1,232), Group 3 (6–12 months, n = 399), and Group 4 (>12 months, n = 366). The patient selection process is detailed in **Figure 1**. This study was approved by the Institutional Review Board of our hospital (Approval No.: 2024-153) and registered with the Chinese Clinical Trial Registry (Registration No.: ChiCTR2400094796).

### 2.2 Endometrial preparation protocols

Hormone replacement therapy (HRT) cycles were used for patients with irregular menstrual cycles. Estrogen supplementation began on days 3–5 of the menstrual cycle, using either a fixed or a gradually increasing dosage. Embryo transfer was scheduled once estradiol levels exceeded 200 pg/mL and endometrial thickness reached ≥7 mm. Subsequently, human chorionic gonadotropin (hCG, 2000 IU) was administered. Progesterone supplementation was initiated the following day to initiate endometrial transformation. FET was performed on the fourth day of progesterone treatment.

Natural cycles were selected for patients with regular ovulation. Transvaginal ultrasound monitoring commenced from days 10–12 of the menstrual cycle to assess follicular growth and endometrial thickness. Upon confirming dominant follicle maturity and optimal endometrial conditions, ovulation was triggered with either hCG (4000 IU) or a gonadotropin-releasing hormone agonist (GnRH-a, 0.2 mg). Progesterone supplementation began two days later, and FET was conducted on the third day of progesterone administration.

Mild stimulation cycles were suitable for selected patients with mild ovulatory disorders who preferred an endometrial environment similar to a natural cycle. Oral ovulation-inducing medications were initiated on days 3–5 of menstruation to stimulate single follicle development and support endometrial growth. Ultrasound monitoring was performed to confirm follicular maturation and an endometrial thickness ≥7 mm. Ovulation was then induced with hCG or GnRH-a, and embryo transfer was performed three days after ovulation.

### 2.3 VF and thawing

VF was performed with a commercial kit (VT101, Kitazato) according to the manufacturer’s guidelines. Briefly, embryos were first equilibrated in an Equilibrium Solution for 12–15 minutes. Next, embryos were transferred to a Vitrification Solution (VS) for 45–60 seconds. Finally, embryos were loaded onto a Cryotop tip with a minimal volume of VS and rapidly plunged into liquid nitrogen for storage.

Embryo thawing was conducted using a warming kit (VT102, Kitazato Corporation). Briefly, embryos on the Cryotop were immediately immersed in a Thawing Solution (TS) for 1 minute. Embryos were then transferred to a Dilution Solution (DS) for 3 minutes, followed by washing in Washing Solution 1 (WS1) for 5 minutes. Subsequently, embryos were placed in Washing Solution 2 (WS2) for 5 minutes. Finally, embryos were transferred into culture medium and incubated for at least 3 hours before transfer.

### 2.4 Outcome measures and assessments

Baseline patient characteristics analyzed included age, body mass index (BMI), infertility duration, type and cause of infertility, basal follicle-stimulating hormone (bFSH) and basal luteinizing hormone (bLH) levels, number of prior implantation failures, number of embryos transferred, number of high-quality embryos, and endometrial preparation protocol. Embryo grading on day 3 of culture followed the Istanbul consensus (16). Grade I embryos were categorized as high-quality. Embryos graded I–III were considered transferable and cryopreserved.

Primary outcomes included clinical pregnancy rate (CPR) and live birth rate (LBR). Secondary outcomes encompassed biochemical pregnancy rate (BPR), multiple pregnancy rate, ectopic pregnancy rate, miscarriage rate, and perinatal outcomes.

Clinical pregnancy was confirmed by ultrasound detection of a gestational sac approximately five weeks after embryo transfer. Live birth was defined as the delivery of a live infant after 28 gestational weeks. Biochemical pregnancy was identified by a positive serum hCG (≥25 IU/L) test performed 14 days after embryo transfer. Multiple pregnancy was confirmed by the ultrasound observation of more than one intrauterine gestational sac. Ectopic pregnancy was confirmed by ultrasound or laparoscopy, identifying at least one gestational sac outside the uterine cavity. Miscarriage was defined as the loss of fetal cardiac activity prior to 20 weeks’ gestation. Perinatal outcomes were assessed only in women delivering singletons. These outcomes included gestational age, preterm birth (<37 weeks), early preterm birth (<32 weeks), birth weight, small for gestational age (SGA), appropriate for gestational age (AGA), large for gestational age (LGA), mode of delivery, and neonatal sex. Classification of SGA and LGA followed Chinese population-based birth weight references (17). Birth weights below the 10th percentile were considered SGA, while those above the 90th percentile indicated LGA. AGA infants had birth weights between the 10th and 90th percentiles.

### 2.5 Statistical analysis

Statistical analyses were conducted using SPSS (version 26.0). Continuous variables were tested for normality using histograms and the Shapiro-Wilk test. Normally or approximately normally distributed data were expressed as mean ± standard deviation (SD) and analyzed using one-way ANOVA. Non-normally distributed data were presented as median (P25, P75) and analyzed using the Kruskal-Wallis test. Categorical variables were expressed as frequency (percentage) and analyzed using the Pearson chi-square test. Statistical significance was defined as P ≤ 0.05.

PSM was applied to minimize baseline differences among groups, fully utilizing available data. Matching covariates included patient age, infertility duration, infertility type and cause, endometrial preparation protocol, number of prior embryo transfer failures, BMI, bFSH, bLH, endometrial thickness on transfer day, number of embryos transferred, and number of high-quality embryos transferred.

Briefly, the propensity scores for each sample were estimated using the mnps function, allowing calculation of the average treatment effect (ATE) and the average weights and sample size for each group. Data were categorized into distinct groups and sorted by weight in descending order. Subsequently, based on respective sample sizes, the corresponding number of samples with the highest weights was selected from each group. After PSM, these selected samples were merged to form a matched dataset.

PSM analyses were performed using SPSS 26.0, R software (version 4.0.4; http://www.R-project.org, The R Foundation), and Statsape software (version BS2.0). Logistic regression models were further applied to evaluate the impact of storage duration on pregnancy and perinatal outcomes.

Furthermore, to ensure the robustness of our findings and address potential concerns regarding the PSM approach, a sensitivity analysis was conducted. We performed multivariable logistic and linear regression analyses on the entire original unmatched cohort (N = 4205). These comprehensive models adjusted for a wide range of clinically relevant covariates, including female age, BMI, infertility duration, infertility type, cause of infertility, bFSH, bLH, number of previous implantation failures, number of embryos transferred, number of good-quality embryos, and endometrial preparation regimen.

## 3 Results

**Figure 2:**
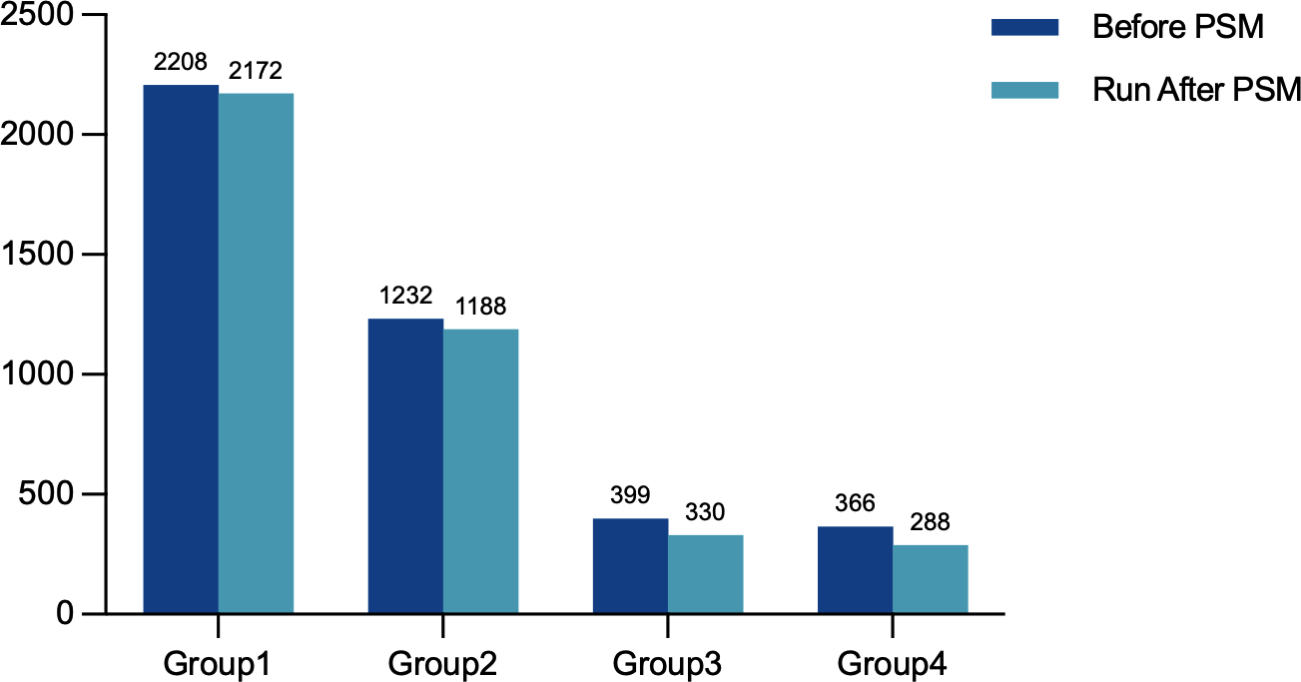
Flowchart of the study cohort before and after matching.

**Figure 3:**
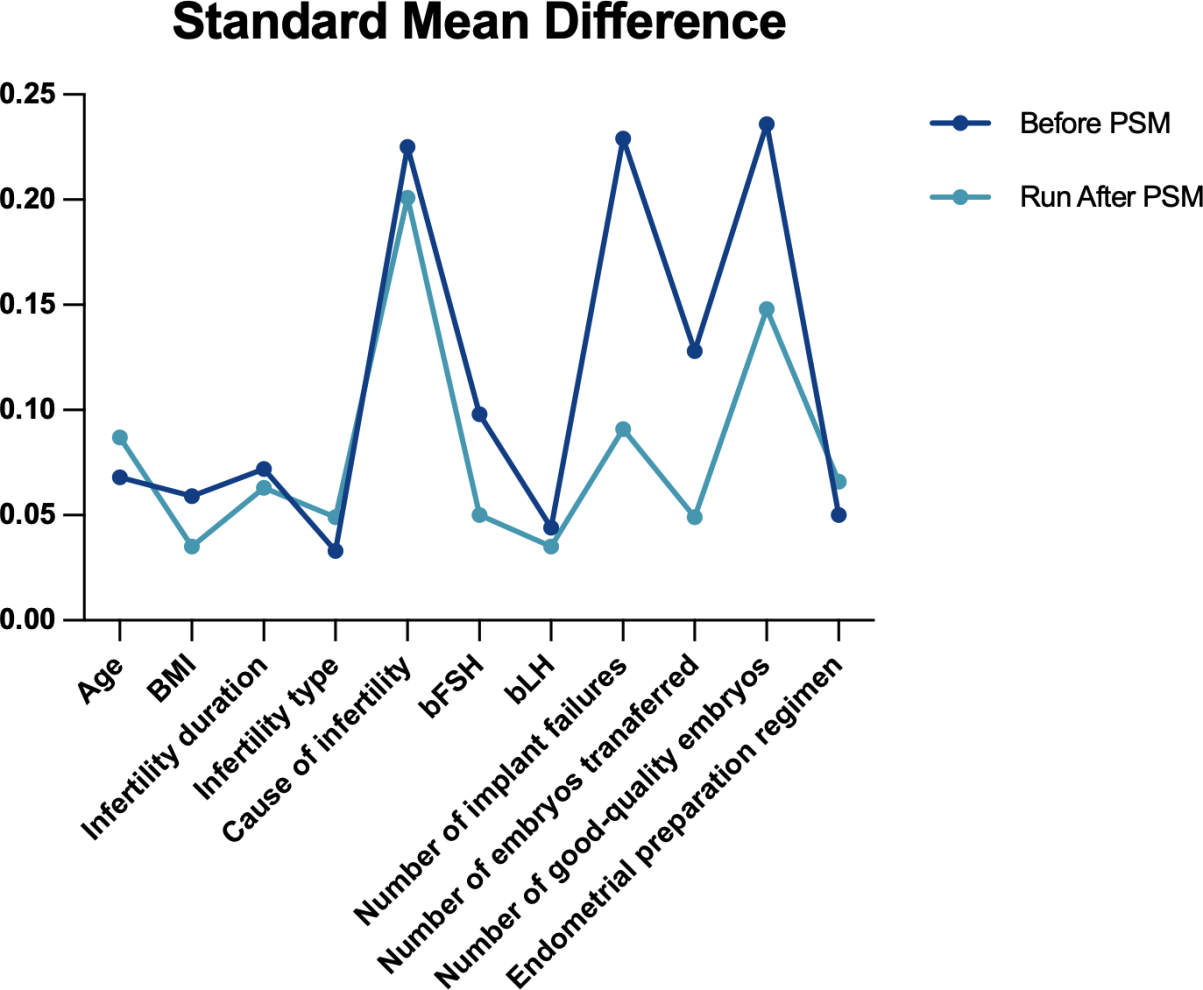
Comparison of SMDs before and after matching.

Initially, this study included 5,234 FET cycles (G1: 2,208 cycles; G2: 1,232 cycles; G3: 399 cycles; G4: 366 cycles). After multi-group PSM, the final matched cohort comprised 2,172 cycles in G1, 1,188 in G2, 330 in G3, and 288 in G4 (**Figure 2**). The standardized mean differences (SMDs) for all covariates before and after matching are displayed in **Figure 3**.

### 3.1 Baseline characteristics before and after PSM

**Table 1** summarizes the baseline characteristics across groups before and after PSM. Before matching, significant differences existed for several covariates, including infertility duration (P = 0.007), infertility causes (P = 0.001), and bFSH levels (P = 0.017). After matching, baseline characteristics were adequately balanced among groups. Most differences became negligible and statistically non-significant (P > 0.05), except for the number of high-quality embryos transferred (P = 0.024).

**Table 1:** Baseline patient characteristics before and after PSM.

Characteristics	Before PSM				P-value	After PSM				P-value
	1 (N = 2208)	2 (N = 1232)	3 (N = 399)	4 (N = 366)		1 (N = 2172)	2 (N = 1188)	3 (N = 330)	4 (N = 288)	
Storage time (months)	0-3	3-6	6-12	>12		0-3	3-6	6-12	>12	
Female age (year)	32(29,35)	32(29,35)	32(29,37)	32(29,36)	0.115	32(29,35)	32(29,35)	32(29,36.25)	32(29,36)	0.078
BMI (kg/m^2^)	23.1(20.8,25.68)	23(20.83,25.7)	23.3(21.3,25.7)	23(20.88,25.9)	0.689	23.1(20.8,25.6)	23(20.9,25.7)	23.4(21.2,25.7)	23.05(21.13,25.8)	0.779
Infertility duration (year)	3(2,4)	3(2,4)	3(2,5)	3(2,5)	0.007	3(2,4)	3(2,4)	3(2,5)	3(2,5)	0.067
Infertility type (%, n)					0.726					0.538
Primary infertility	47.15 (1041/2208)	47.81 (589/1232)	45.36 (181/399)	45.08 (165/366)		47.05 (1022/2172)	47.56 (565/1188)	44.85 (148/330)	43.40 (125/288)	
Secondary infertility	52.85 (1167/2208)	52.19 (643/1232)	54.64 (218/399)	54.92 (201/366)		52.95 (1150/2172)	52.44 (623/1188)	55.15 (182/330)	56.60 (163/288)	
Cause of infertility (%, n)					0.001					0.102
Tubal factor	69.52 (1535/2208)	66.64 (821/1232)	68.92 (275/399)	68.58 (251/366)		69.57 (1511/2172)	68.01 (808/1188)	69.70 (230/330)	70.49 (203/288)	
PCOS	14.63 (323/2208)	17.04 (210/1232)	14.28 (57/399)	16.12 (59/366)		14.55 (316/2172)	17.17 (204/1188)	15.76 (52/330)	14.93 (43/288)	
Endometriosis	3.71 (82/2208)	3.08 (38/1232)	2.26 (9/399)	5.46 (20/366)		3.68 (80/2172)	3.11 (37/1188)	1.52 (5/330)	4.86 (14/288)	
Male	4.62 (102/2208)	3.41 (42/1232)	5.76 (23/399)	6.56 (24/366)		4.65 (101/2172)	3.45 (41/1188)	5.15 (17/330)	6.25 (18/288)	
Couple	6.29 (139/2208)	7.39 (91/1232)	6.77 (27/399)	2.19 (8/366)		6.31 (137/2172)	6.99 (83/1188)	6.67 (22/330)	2.43 (7/288)	
DOR	0.91 (20/2208)	1.87 (23/1232)	2.01 (8/399)	1.09 (4/366)		0.92 (20/2172)	1.09 (13/1188)	1.20 (4/330)	1.04 (3/288)	
Unknown	0.32 (7/2208)	0.57 (7/1232)	0	0		0.32 (7/2172)	0.18 (2/1188)	0	0	
bFSH (IU/L)	6.82(5.63,8.05)	6.90(5.65,8.15)	7.22(5.82,8.54)	7.13(5.68,8.09)	0.017	6.85(5.65,8.06)	6.89(5.64,8.12)	7.07(5.76,8.28)	7.16(5.87,8.18)	0.168
bLH (IU/L)	4.8(3.33,6.66)	4.99(3.52,6.67)	4.62(3.14,6.72)	4.62(3.23,6.15)	0.07	4.8(3.35,6.62)	4.97(3.53,6.64)	4.64(3.27,6.66)	4.65(3.36,6.22)	0.258
Number of implant failures (%, n)					< 0.001					0.068
≤ 1	96.92 (2140/2208)	96.02 (1183/1232)	92.73 (370/399)	85.79 (314/366)		96.87 (2104/2172)	96.21 (1143/1188)	94.24 (311/330)	97.57 (281/288)	
≥ 2	3.08 (68/2208)	3.98 (49/1232)	7.27 (29/399)	14.21 (52/366)		3.13 (68/2172)	3.79 (45/1188)	5.76 (19/330)	2.43 (7/288)	
Number of embryos transferred (%, n)					< 0.001					0.274
1	8.65 (191/2208)	10.71 (132/1232)	16.29 (65/399)	8.74 (32/366)		8.79 (191/2172)	10.52 (125/1188)	11.21 (37/330)	9.03 (26/288)	
2	91.35 (2017/2208)	89.29 (1100/1232)	83.71 (334/399)	91.26 (334/366)		91.21 (1981/2172)	89.48 (1063/1188)	88.79 (293/330)	90.97 (262/288)	
Number of good-quality embryos (%, n)					< 0.001					0.024
0	37.09 (819/2208)	39.61 (488/1232)	45.61 (182/399)	35.52 (130/366)		37.38 (812/2172)	39.48 (469/1188)	43.33 (143/330)	39.24 (113/288)	
1	50.54 (1116/2208)	48.05 (592/1232)	46.12 (184/399)	41.26 (151/366)		50.23 (1091/2172)	47.98 (570/1188)	48.18 (159/330)	43.75 (126/288)	
2	12.37 (273/2208)	12.34 (152/1232)	8.27 (33/399)	23.22 (85/366)		12.39 (269/2172)	12.54 (149/1188)	8.49 (28/330)	17.01 (49/288)	
Endometrial preparation regimen (%, n)					0.683					0.458
Natural cycle	35.19 (777/2208)	32.47 (400/1232)	35.34 (141/399)	36.07 (132/366)		35.31 (767/2172)	31.90 (379/1188)	33.64 (111/330)	37.15 (107/288)	
Replacement cycle	57.02 (1259/2208)	58.85 (725/1232)	55.89 (223/399)	56.28 (206/366)		56.95 (1237/2172)	59.34 (705/1188)	58.48 (193/330)	54.52 (157/288)	
Mild stimulation cycle	7.79 (172/2208)	8.68 (107/1232)	8.77 (35/399)	7.65 (28/366)		7.74 (168/2172)	8.76 (104/1188)	7.88 (26/330)	8.33 (24/288)	

PSM, propensity score matching; BMI, body mass index; PCOS, Polycystic Ovary Syndrome; DOR, Diminished Ovarian Reserve; bFSH, basal follicle-stimulating hormone; bLH, basal luteinizing hormone.

### 3.2 Pregnancy outcomes

Given the significant baseline imbalances prior to PSM, the initial outcome comparisons were considered confounded. After adjusting for confounding factors through PSM, intergroup differences substantially changed. **Table 2** illustrates pregnancy outcomes for FET cycles stratified by storage duration. The BPR, CPR, and LBR all declined with increasing storage time. Specifically, BPR was 48.53% for storage durations under three months, decreasing to 40.28% for durations exceeding twelve months. CPR declined from 48.20% in G1 to 40.28% in G4. Similarly, LBR dropped from 39.46% in G1 to 29.17% in G4. Conversely, the miscarriage rates increased from 15.95% in G1 to 24.14% in G4.

**Table 2:** Comparison of pregnancy outcomes before and after PSM.

Outcomes	Before PSM				P-value			After PSM				P-value		
	1 (N = 2208)	2 (N = 1232)	3 (N = 399)	4 (N = 366)	P1	P2	P3	1 (N = 2172)	2 (N = 1188)	3 (N = 330)	4 (N = 288)	P1	P2	P3
Storage time (months)	0-3	3-6	6-12	>12				0-3	3-6	6-12	>12			
Biochemical pregnancy rate (%, n)	48.69 (1075/2208)	44.56 (549/1232)	42.86 (171/399)	43.99 (161/366)	0.02	0.032	0.096	48.53 (1054/2172)	45.20 (537/1188)	43.33 (143/330)	40.28 (116/288)	0.065	0.078	0.008
Clinical pregnancy rate (%, n)	48.37 (1068/2208)	43.91 (541/1232)	42.61 (170/399)	43.72 (160/366)	0.012	0.034	0.099	48.20 (1047/2172)	44.95 (534/1188)	43.03 (142/330)	40.28 (116/288)	0.071	0.079	0.011
Ectopic pregnancy rate(%, n)	1.18 (26/2208)	1.38 (17/1232)	1.00 (4/399)	1.64 (6/366)	0.609	0.763	0.46	1.06 (23/2172)	1.52 (18/1188)	0.91 (3/330)	1.39 (4/288)	0.25	0.803	0.614
Pregnancy loss rate (%, n)	15.82 (169/1068)	18.85 (102/541)	24.71 (42/170)	26.25 (42/160)	0.125	0.004	0.001	15.95 (167/1047)	19.48 (104/534)	23.94 (34/142)	24.14 (28/116)	0.079	0.017	0.025
Live birth rate (%, n)	39.54 (873/2208)	34.25 (422/1232)	31.08 (124/399)	30.60 (112/366)	0.002	0.001	0.001	39.46 (857/2172)	34.68 (412/1188)	31.82 (105/330)	29.17 (84/288)	0.006	0.008	0.001
Multiple pregnancy rate (%, n)	24.63 (215/873)	27.73 (117/422)	22.58 (28/124)	12.50 (14/112)	0.232	0.619	0.004	24.62 (211/857)	28.16 (116/412)	20.95 (22/105)	13.10 (11/84)	0.178	0.408	0.018

P: G1 vs. G2 vs. G3 vs. G4, P1: G2 vs. G1, P2:G3 vs. G1, P3: G4 vs. G1

PSM, propensity score matching.

Given residual covariate imbalance post-PSM, multivariate logistic regression analyses were performed (**Table 3**). Results indicated that prolonged embryo storage was independently associated with lower BPR, CPR, and LBR, even after adjusting for the number of high-quality embryos transferred. LBR significantly decreased when storage durations exceeded three months, whereas significant reductions in BPR and CPR were observed only after storage exceeded twelve months. Conversely, miscarriage rates significantly increased after six months of embryo storage.

**Table 3:** aORs for pregnancy outcomes by duration of vitrified embryo storage.

Outcomes	G2					G3					G4				
	B^a^	Standard error	OR	OR 95%CI	P-value	B^a^	Standard error	OR	OR 95%CI	P-value	B^a^	Standard error	OR	OR 95%CI	P-value
Biochemical pregnancy rate	-0.125	0.074	0.883	0.764-1.02	0.09	-0.165	0.121	0.848	0.669-1.075	0.173	-0.349	0.13	0.705	0.547-0.91	0.007
Clinical pregnancy rate	-0.122	0.074	0.885	0.766-1.022	0.097	-0.165	0.121	0.848	0.669-1.075	0.173	-0.336	0.13	0.715	0.554-0.922	0.01
Ectopic pregnancy rate	0.367	0.317	1.444	0.776-2.687	0.247	-0.138	0.617	0.871	0.26-2.92	0.823	0.274	0.546	1.316	0.451-3.836	0.615
Pregnancy loss rate	0.237	0.139	1.268	0.967-1.663	0.087	0.502	0.214	1.652	1.085-2.515	0.019	0.515	0.233	1.674	1.06-2.643	0.027
Live birth rate	-0.198	0.076	0.82	0.707-0.952	0.009	-0.297	0.127	0.743	0.579-0.954	0.02	-0.472	0.138	0.624	0.476-0.818	0.001
Multiple pregnancy rate	0.229	0.137	1.257	0.962-1.643	0.094	-0.162	0.254	0.851	0.517-1.4	0.524	-0.817	0.335	0.442	0.229-0.852	0.015

a Adjusted for number of good-quality embryos.

To further validate these findings, a sensitivity analysis using multivariable logistic regression on the entire unmatched cohort (N = 4205) was performed, adjusting for comprehensive baseline characteristics including maternal age, previous implantation failures, and endometrial preparation method. The results remained consistent with the PSM analysis, confirming that prolonged storage duration is independently associated with poorer pregnancy outcomes (**Supplementary Table 1**).

### 3.3 Perinatal outcomes

Perinatal outcomes were analyzed exclusively in women who delivered singletons, resulting in 1,157 cycles available for comparison. Significant baseline differences existed before PSM. Therefore, the initial perinatal outcome comparisons were potentially biased. After adjusting for baseline confounders, intergroup differences notably changed (**Table 4**). Prolonged storage significantly decreased neonatal gestational age, with median gestational age declining from 38 weeks in G1 to 37 weeks in G4.

**Table 4:** Comparison of perinatal outcomes before and after PSM.

Outcomes	Before PSM				P-value			After PSM					P-value		
	1 (N = 2208)	2 (N = 1232)	3 (N = 399)	4 (N = 366)	P1	P2	P3	1 (N = 2172)	2 (N = 1188)	3 (N = 330)	4 (N = 288)	P1		P2	P3
Storage time (months)	0-3	3-6	6-12	>12				0-3	3-6	6-12	>12				
Newborn gender(%,n)					0.286	0.126	0.712					0.315		0.161	0.935
Female	48.94 (322/658)	45.25 (138/305)	57.29 (55/96)	46.94 (46/98)				48.45 (313/646)	44.93 (133/296)	56.63 (47/83)	47.95 (35/73)				
Male	51.06 (336/658)	54.75 (167/305)	42.71 (41/96)	53.06 (52/98)				51.55 (333/646)	55.07 (163/296)	43.37 (36/83)	52.05 (38/73)				
Gestational age(weeks)	38 (37,39)	38 (37,39)	38 (36,39)	37 (36,38.25)	0.358	0.173	0.001	38 (37,39)	38 (37,39)	38 (36,39)	37 (36,38.5)	0.053		0.026	0.003
Preterm (<37weeks) (%,n)	15.05 (99/658)	18.69 (57/305)	30.21 (29/96)	29.59 (29/98)	0.153	< 0.001	< 0.001	15.02 (97/646)	18.58 (55/296)	31.33 (26/83)	26.03 (19/73)	0.167		< 0.001	0.015
Cesarean section rate(%,n)	72.19 (475/658)	72.46 (221/305)	73.96 (71/96)	71.43 (70/98)	0.93	0.717	0.876	72.14 (466/646)	71.96 (213/296)	74.70 (62/83)	67.12 (49/73)	0.955		0.623	0.368
Birth weight(g)	3400(3100,3700)	3400(3100,3800)	3300(3000,3600)	3360(3100,3627.5)	0.578	0.017	0.376	3400(3100,3702.5)	3425(3112.5,3790)	3280(3000,3600)	3350(3100,3685)	0.627		0.011	0.543
Birth length(cm)	50 (50,51)	50 (50,51)	50 (49,51)	50 (48,50)	0.947	0.082	0.008	50 (50,51)	50 (50,51)	50 (49,51)	50 (49.5,50)	0.868		0.059	0.034
SGA rate(%,n)	2.28 (15/658)	3.28 (10/305)	3.13 (3/96)	2.04 (2/98)	0.364	0.612	0.882	2.32 (15/646)	2.70 (8/296)	3.61 (3/83)	2.74 (2/73)	0.725		0.475	0.824
AGA rate(%,n)	57.45 (378/658)	50.82 (155/305)	62.50 (60/96)	48.98 (48/98)	0.054	0.349	0.115	56.97 (368/646)	51.69 (153/296)	62.65 (52/83)	49.32 (36/73)	0.13		0.324	0.212
LGA rate(%,n)	40.27 (265/658)	45.90 (140/305)	34.38 (33/96)	48.98 (48/98)	0.1	0.269	0.103	40.71 (263/646)	45.61 (135/296)	33.73 (28/83)	47.95 (35/73)	0.158		0.222	0.234

P: G1 vs. G2 vs. G3 vs. G4,P1: G2 vs. G1, P2:G3 vs. G1,P3:G4 vs. G1

PSM, propensity score matching; SGA, small for gestational age; AGA, appropriate for gestational age; LGA, large for gestational age.

Residual covariate imbalance after PSM required further adjustment through multivariate logistic and linear regression analyses. Unadjusted and adjusted results are presented in **Tables 5**, **6**. Our analysis revealed neonatal gestational age decreased significantly after embryo storage surpassed six months. This association remained significant even after adjusting for the number of high-quality embryos transferred.

**Table 5:** aORs for categorical perinatal outcomes by duration of vitrified embryo storage.

Outcomes	G2					G3					G4				
	B^a^	Standard error	OR	OR 95%CI	P-value	B^a^	Standard error	OR	OR 95%CI	P-value	B^a^	Standard error	OR	OR 95%CI	P-value
Preterm (<37weeks)	0.253	0.187	1.287	0.893-1.857	0.176	0.959	0.262	2.608	1.56-4.362	< 0.001	0.622	0.291	1.863	1.052-3.298	0.033
Cesarean section rate	-0.025	0.157	0.975	0.717-1.327	0.873	0.119	0.268	1.126	0.666-1.903	0.658	-0.231	0.266	0.794	0.472-1.337	0.386
SGA rate	0.147	0.445	1.158	0.484-2.772	0.741	0.44	0.645	1.553	0.439-5.493	0.495	0.265	0.767	1.303	0.29-5.855	0.73
AGA rate	-0.221	0.142	0.802	0.607-1.058	0.119	0.227	0.241	1.254	0.782-2.012	0.347	-0.26	0.249	0.771	0.473-1.256	0.296
LGA rate	0.209	0.142	1.232	0.932-1.629	0.142	-0.288	0.246	0.75	0.463-1.215	0.243	0.237	0.25	1.267	0.777-2.067	0.343

a Adjusted for number of good-quality embryos.

SGA, small for gestational age; AGA, appropriate for gestational age; LGA, large for gestational age.

**Table 6:** Adjusted coefficients for continuous perinatal outcomes by duration of vitrified embryo storage.

Outcomes	G2			G3			G4		
	B^a^	Standard error	P-value	B^a^	Standard error	P-value	B^a^	Standard error	P-value
Gestational age	-0.228	0.15	0.128	-0.564	0.249	0.023	-0.701	0.264	0.008
Birth weight	31.044	39.718	0.435	-177.018	65.808	0.007	-33.42	69.962	0.633
Birth length	-0.102	0.199	0.609	-0.581	0.33	0.078	-0.591	0.35	0.092

a Adjusted for number of good-quality embryos.

Similarly, the sensitivity analysis on the unmatched cohort supported the post-PSM findings, showing a consistent trend regarding perinatal outcomes, particularly the reduction in gestational age with prolonged storage (**Supplementary Tables 2**, **3**).

## 4 Discussion

This study included 4,205 FET cycles that met the inclusion criteria. To minimize potential confounding, we employed multi-group PSM. The final analysis comprised 3,978 cycles for pregnancy outcomes and 1,157 for perinatal outcomes. After adjustment, we found that prolonged storage was associated with a significant decline in LBR after three months, an increase in miscarriage rate after six months, and a reduction in BPR and CPR beyond twelve months. Furthermore, neonatal gestational age was significantly shorter when storage exceeded six months.

VF is now a cornerstone of ART, and the techniques for embryo cryopreservation have advanced considerably. With the increasing utilization of frozen cycles and extended storage durations, concerns regarding the long-term safety of VF have garnered significant attention. Existing literature on prolonged embryo storage presents conflicting results. Animal studies by Mozdarani et al. reported decreased survival rates and increased chromosomal abnormalities in mouse embryos with extended storage durations (18). Conversely, other animal studies found no significant effects of VF storage duration on embryo survival, pregnancy outcomes, or LBRs (19–22). In human studies, early research by Testart et al. indicated decreased embryo survival after several months of storage (23).

In contrast, a retrospective study by Riggs et al. concluded that storage time did not influence survival rates in patients using autologous or donor oocytes (11). Likewise, Liu et al. (13) and Yuan et al. (24) observed no association between storage duration and embryo survival. However, these earlier studies primarily relied on slow-freezing methods, limiting the generalizability of their findings to contemporary VF techniques.

Recent human studies on VF storage time also yield inconsistent conclusions. Li et al. suggested that pregnancy and neonatal outcomes were unaffected by storage duration (25). However, substantial baseline differences and unadjusted confounders in that study may compromise the reliability of its conclusions. Ma et al. conducted a retrospective analysis of 2,938 vitrified blastocyst-transfer cycles (26). They reported no significant adverse effects of extended storage on embryos or offspring. Nevertheless, their study was restricted to single, high-quality blastocyst transfers, which may limit its broader applicability. In contrast, Zhan et al. found that storage beyond five years reduced implantation and live birth rates in FET cycles involving high-quality blastocysts (27). This finding is supported by Cui et al., who observed significantly lower clinical pregnancy and live birth rates after storage exceeding five years (28). Li et al. also reported that long-term storage adversely affected pregnancy outcomes, decreasing biochemical pregnancy, clinical pregnancy, and live birth rates, although neonatal outcomes were comparable (10). Most similar studies relied solely on multivariate regression analyses. In contrast, PSM reduces selection bias and simplifies statistical adjustment, making it particularly suitable for observational research. Among studies employing PSM, most compared only two storage groups, typically <1 year versus >5 years. In this context, our study is the first to apply multi-group PSM specifically to vitrified cleavage-stage embryos in FET cycles. Our findings thereby provide important additional evidence.

A primary concern in evaluating embryo storage duration is confounding by indication, wherein delayed transfer merely reflects underlying clinical challenges rather than the effect of storage time itself. For instance, longer storage may reflect the need to treat severe pelvic pathologies (e.g., endometriosis) or prior transfer failures. However, our study rigorously addressed this bias utilizing our comprehensive dataset. By employing multi-group PSM, we successfully balanced key indicators of clinical complexity across all groups, explicitly including the cause of infertility, infertility duration, and the number of prior implantation failures (**Table 1**). Furthermore, our sensitivity analysis on the unmatched cohort, which comprehensively adjusted for these clinical variables, yielded consistently poorer outcomes with extended storage. Consequently, because the decline in clinical outcomes persists even when comparing patients with similar baseline clinical complexities, our data strongly suggest an independent association with prolonged cryopreservation.

Theoretically, cellular biological activity and metabolism are completely halted at the temperature of liquid nitrogen (-196 °C), suggesting that storage duration itself should not directly impair embryo viability through active metabolic processes. However, the impact of the prolonged storage environment requires careful consideration. In modern standardized IVF laboratories, physical environmental fluctuations—such as transient temperature changes from accessing liquid nitrogen tanks—are tightly controlled and likely negligible. Instead, the primary concern within this storage environment is chemical. Vitrified embryos are continuously exposed to the extremely high concentrations of cryoprotectants required for the vitrification process. We hypothesize that prolonged exposure to this specific chemical environment might exert undetected epigenetic or cytotoxic effects over long periods, potentially disrupting early developmental processes upon thawing. These potential long-term chemical impacts of the storage environment on embryo integrity warrant further molecular investigation.

Our results indicated a significant reduction in neonatal gestational age when storage exceeded six months. Previous studies evaluating perinatal outcomes reported a non-significant trend toward increased risk of low birth weight with longer storage after adjusting for confounders. Zhan et al. observed no significant effects of VF storage duration on preterm birth, birth weight, or sex ratio (27). However, they reported an increased proportion of LGA and a decreased proportion of SGA infants with prolonged storage. Mao et al. found no significant differences in birth weight, height, or congenital anomalies associated with longer storage times (29).

In contrast, our study demonstrated a decline in gestational age with prolonged storage. We detected no significant changes in LGA or SGA proportions. Additionally, preterm birth rates did not consistently rise with storage duration. Therefore, the primary risk associated with prolonged storage might pertain to child development, such as delayed physical growth. These potential long-term impacts necessitate further follow-up (30). Overall, long-term embryo storage did not demonstrate clear negative effects on most perinatal outcomes, which provides reassuring evidence regarding the safety of extended embryo storage.

Although this study was limited to cleavage-stage embryos, our findings establish a relationship between longer storage and reduced pregnancy rates alongside increased miscarriage rates. These results may inform clinical decision-making and laboratory practices in ART. Clinicians should consider storage duration when scheduling embryo transfers. Future research should focus on elucidating the mechanisms underlying the decline in outcomes and on refining VF techniques. With the growing demand for fertility preservation, concerns regarding the long-term safety of embryo storage have become prominent. Our observation that extended storage decreases LBRs and increases miscarriage rates provides crucial risk-benefit information for patients postponing childbirth for medical or personal reasons.

Furthermore, this retrospective study identified an association between prolonged embryo storage and reduced gestational age. Ensuring the safety of long-term VF requires extended follow-up studies evaluating both physiological and psychological health in offspring. As frozen embryo transfer becomes increasingly prevalent, this research contributes valuable evidence regarding the impact of storage duration on pregnancy outcomes. Additionally, it offers new insights into discussions about recommended upper limits for vitrified embryo storage.

This study has several limitations that should be explicitly acknowledged. Primarily, its retrospective design inherently makes it susceptible to selection bias and unmeasured confounding. Although we utilized rigorous PSM and conducted a comprehensive sensitivity analysis on the unmatched cohort to adjust for known clinical variables (such as prior implantation failures and endometrial preparation methods), we lacked detailed data on the specific clinical, surgical, or personal reasons dictating the delay in embryo transfer. Consequently, we cannot completely rule out confounding by indication—specifically, the possibility that embryos stored for longer periods belong to patients with more complex, refractory reproductive challenges that independently lower success rates. Future prospective studies incorporating detailed patient timelines, reasons for transfer delay, and cumulative live birth rates are essential to definitively disentangle the effect of storage time from underlying patient pathology. Furthermore, as this study reflects the practices of a single center, our findings warrant external validation in multicenter cohorts.

## 5 Conclusion

This retrospective analysis of 4,205 vitrified cleavage-stage embryo transfer cycles revealed that prolonged cryopreservation duration is associated with poorer pregnancy and perinatal outcomes. Specifically, the LBR began to decline significantly after storage exceeded 3 months, while the miscarriage rate increased markedly beyond 6 months of storage. Moreover, both BPR and CPR decreased significantly when storage extended beyond 12 months. Additionally, storage for more than 6 months was correlated with a significant reduction in neonatal gestational age. These findings suggest that, although VF has become a routine procedure in assisted reproduction, long-term storage may exert cumulative adverse effects on embryonic potential. Storage duration should be considered in clinical decision-making, and FET should be scheduled as early as feasible. Future studies with larger sample sizes and longer follow-up are warranted to validate the safety limits of storage duration further and elucidate the underlying mechanisms.

## Data Availability

The raw data supporting the conclusions of this article will be made available by the authors, without undue reservation.
