# Supplementary material for: Impact of post-vitrification storage duration on clinical outcomes of frozen-thawed cleavage-stage embryos transfer: a propensity score-matched retrospective cohort study
Source: Front Endocrinol (Lausanne). 2026 Apr 2;17:1765681. doi: 10.3389/fendo.2026.1765681 (PMC13082996; doi:10.3389/fendo.2026.1765681)
Supplement: Supplementary file 1 [file DataSheet1.pdf]

Supplementary Table 1 aORs for pregnancy outcomes by duration of vitrified embryo storage

	G2					G3					G4				
	B[a]	Standard error	OR	OR 95% CI	P-value	B[a]	Standard error	OR	OR 95% CI	P-value	B[a]	Standard error	OR	OR 95% CI	P-value
Biochemical pregnancy rate	-0.166	0.075	0.847	0.731-0.982	0.028	-0.104	0.117	0.901	0.717-1.133	0.373	-0.219	0.121	0.803	0.634-1.018	0.070
Clinical pregnancy rate	-0.181	0.075	0.834	0.720-0.967	0.016	-0.102	0.117	0.903	0.719-1.135	0.384	-0.214	0.121	0.808	0.637-1.024	0.077
Ectopic pregnancy rate	0.152	0.317	1.164	0.626-2.165	0.631	-0.140	0.545	0.869	0.299-2.531	0.798	0.170	0.478	1.185	0.464-3.023	0.723
Pregnancy loss rate	0.183	0.141	1.201	0.911-1.583	0.194	0.360	0.205	1.434	0.960-2.141	0.078	0.449	0.209	1.567	1.040-2.359	0.032
Live birth rate	-0.229	0.078	0.795	0.638-0.925	0.003	-0.243	0.122	0.785	0.617-0.997	0.048	-0.382	0.128	0.683	0.531-0.877	0.003
Multiple pregnancy rate	0.234	0.138	1.264	0.964-1.658	0.090	0.014	0.236	1.014	0.638-1.610	0.954	-0.836	0.308	0.434	0.237-0.793	0.007

[a]Adjusted for female age, BMI, infertility duration, infertility type, cause of infertility, bFSH, bLH, number of implant failures, number of embryos transferred, number of good-quality embryos, endometrial preparation regimen.

Supplementary Table 2 aORs for categorical perinatal outcomes by duration of vitrified embryo storage

	G2					G3					G4				
	B[a]	Standard error	OR	OR 95% CI	P-value	B[a]	Standard error	OR	OR 95% CI	P-value	B[a]	Standard error	OR	OR 95% CI	P-value
Preterm (<37weeks)	0.308	0.188	1.361	0.941-1.968	0.101	1.003	0.257	2.727	1.647-4.515	< 0.001	0.685	0.263	1.985	1.185-3.325	0.009
Cesarean section rate	-0.003	0.160	0.997	0.729-1.364	0.985	0.012	0.257	1.012	0.612-1.673	0.963	-0.190	0.255	0.827	0.502-1.363	0.456
SGA rate	0.349	0.420	1.418	0.622-3.232	0.406	0.088	0.664	1.092	0.297-4.015	0.895	-0.068	0.784	0.934	0.201-4.341	0.930
AGA rate	-0.290	0.142	0.748	0.566-0.988	0.041	0.236	0.231	1.267	0.806-1.992	0.306	-0.177	0.229	0.837	0.535-1.311	0.438
LGA rate	0.257	0.143	1.294	0.978-1.712	0.072	-0.261	0.234	0.770	0.486-1.219	0.265	0.189	0.229	1.208	0.771-1.893	0.408

[a]Adjusted for female age, BMI, infertility duration, infertility type, cause of infertility, bFSH, bLH, number of implant failures, number of embryos transferred, number of good-quality embryos, endometrial preparation regimen.

Abbreviations: SGA, small for gestational age; AGA, appropriate for gestational age; LGA, large for gestational age.

Supplementary Table 3 Adjusted coefficients for continuous perinatal outcomes by duration of vitrified embryo storage

	G2			G3			G4		
	B[a]	Standard error	P-value	B[a]	Standard error	P-value	B[a]	Standard error	P-value
Gestational age	-0.233	0.148	0.117	-0.570	0.234	0.015	-0.606	0.238	0.011
Birth weight	37.557	39.554	0.343	-159.229	62.469	0.011	-39.074	63.644	0.539
Birth length	-0.034	0.197	0.863	-0.519	0.312	0.096	-0.454	0.318	0.153

[a]Adjusted for female age, BMI, infertility duration, infertility type, cause of infertility, bFSH, bLH, number of implant failures, number of embryos transferred, number of good-quality embryos, endometrial preparation regimen.
